# Risk Factors of Mortality From *Pneumocystis* Pneumonia in Non-HIV Patients: A Meta-Analysis

**DOI:** 10.3389/fpubh.2021.680108

**Published:** 2021-06-16

**Authors:** Yuqiong Wang, Xiaoyi Zhou, Maidinuer Saimi, Xu Huang, Ting Sun, Guohui Fan, Qingyuan Zhan

**Affiliations:** ^1^Peking University China-Japan Friendship School of Clinical Medicine, Beijing, China; ^2^Department of Pulmonary and Critical Care Medicine, Center of Respiratory Medicine, China-Japan Friendship Hospital, Capital Medical University, Beijing, China; ^3^Department of Pulmonary and Critical Care Medicine, Center of Respiratory Medicine, China-Japan Friendship Hospital, Beijing, China; ^4^National Center for Respiratory Medicine, Beijing, China; ^5^Institute of Clinical Medical Sciences, China-Japan Friendship Hospital, Beijing, China; ^6^Institute of Respiratory Medicine, Chinese Academy of Medical Sciences, National Clinical Research Center for Respiratory Disease, National Center for Respiratory Disease, Beijing, China

**Keywords:** *Pneumocystis* pneumonia, non-HIV, mortality, risk factors, pneumonia

## Abstract

**Background:** We performed a meta-analysis to systematically review the risk factors of mortality from non-HIV-related *Pneumocystis* pneumonia (PcP) and provide the theoretical basis for managing non-HIV-related PcP.

**Methods:** PubMed, Embase, Web of Science, the Cochrane Library and CNKI databases were searched. A meta-analysis of the risk factors of mortality from non-HIV-related PcP was conducted.

**Results:** A total of 19 studies and 1,310 subjects were retrieved and included in the meta-analysis, including 485 and 825 patients in the non-survivor and survivor groups, respectively. In the primary analysis, age, concomitant with other pulmonary diseases at diagnosis of PcP, solid tumors, cytomegalovirus(CMV) co-infection, lactate dehydrogenase (LDH), lymphocyte count, invasive ventilation during hospitalization, and pneumothorax were associated with mortality from non-HIV-related PcP, whereas sex, albumin, PcP prophylaxis, use of corticosteroids after admission, and time from onset of symptoms to treatment were not associated with mortality from non-HIV-related PcP.

**Conclusions:** The mortality rate of non-HIV-infected patients with PcP was still high. Age, concomitant with other pulmonary diseases at diagnosis of PcP, solid tumors, CMV co-infection, LDH, lymphocyte count, invasive ventilation during hospitalization, and pneumothorax were risk factors of mortality from non-HIV-related PcP. Improved knowledge of prognostic factors is crucial to guide early treatment.

## Introduction

*Pneumocystis* pneumonia (PcP) is a potentially life-threatening infection that occurs in immunocompromised patients (1). Recent investigations have reported a decrease in PcP incidence in human immunodeficiency virus (HIV) patients due to the advent of highly active antiretroviral therapy (HAART) and chemoprophylaxis (2). Conversely, the incidence of PcP, which has been increasing in immunocompromised patients without HIV during the last decades, has been attributed to the increasing number of patients at risk who are receiving corticosteroids or other immunosuppressive medications for allogeneic bone marrow or organ transplant, malignancies, and autoimmune inflammatory diseases (3). In non-HIV immunocompromised patients with PcP infection, there is a significant risk of morbidity and mortality, and the prognosis is dismal once mechanical ventilation for respiratory failure is required (4). Several studies have attempted to explore the risk factors influencing the mortality in non-HIV patients with PcP. This meta-analysis was performed to explore the risk factors of mortality from PcP in non-HIV patients.

## Materials and Methods

### Search Strategy

A search of PubMed, Embase, Web of Science, the Cochrane Library and CNKI databases were undertaken comprehensively and systematically for case-control and cohort studies published up to July 2020 on risk factors of death in non-HIV patients with PcP. The references were retroactively included to supplement and access relevant literature. The retrieval adopted a method of combining subject words with free words. English search terms included the synonyms of domains (pneumonia, *pneumocystis*) and (non-HIV), determinant (risk factors), and outcome (death).

### Inclusion and Exclusion Criteria

Studies were included if they fulfilled the following criteria: (1) case-control or cohort studies including non-HIV patients with PcP; (2) studies exploring the risk factors of death in-hospital or within 30 days of PcP in non-HIV patients (to ensure the consistency of outcome indicators). The exclusion criteria were defined as follows: (1) patients aged <18 years; (2) repeated publications; (3) defective or poor quality of study; (4) reviews, case reports, meta-analyses, opinions, and summaries.

### Literature Selection and Data Extraction

Two researchers (YW and XZ) independently conducted literature screening, data extraction, and quality evaluation of the included studies. In the literature screening process, the title and abstract were first read. After excluding the literature that did not meet the inclusion criteria, the full text of the remaining literature was read to determine whether it should be included in the meta-analysis. The authors were contacted by email or telephone to supplement where the data were incomplete.

### Study Quality Assessment

The New castle Ottawa Scale (NOS) (5), which is quite comprehensive and has been partially validated for evaluating the quality of observational studies in meta-analyses. Table 1 shows the quality assessment based on the NOS guidelines. High-quality literature was classified as ≥6 points, and most studies were rated as having reasonable to good quality, with a low risk of bias. In some studies, a small number of cases, different definitions of the analyzed risk factors, and dissimilarity of the populations were the main weaknesses. Data extraction and quality assessment were conducted independently by two authors and discussed with a third investigator when there was disagreement. Since this was a review of already published data, written informed consent was waived by the ethics committee.

**Table 1:** Characteristics and Newcastle-Ottawa scale quality score of included studies.

**References**	**Study type**	**NOS**	**Country**	**Number of patients**	**Period**	**Mean age, y**	**Male, (%)**	**Outcome**
Asai et al. (6)	Case-control study	8	Japan	38	2005–2012	NA	NA	30-day mortality
Chen et al. (7)	Case-control study	8	China	69	2004–2013	39	25 (36.23%)	In-hospital mortality
Hardak et al. (8)	Case-control study	8	Israel	58	2005–2010	56	28 (48.3%)	In-hospital mortality
Kageyama et al. (9)	Case-control study	8	Japan	95	2000–2015	69	57 (60%)	In-hospital mortality
Kim et al. (10)	Case-control study	6	Korea	173	2004–2011	56	116(67.05%)	In-hospital mortality
Kim et al. (11)	Cohort study	7	Korea	76	2014–2015	55	53 (65%)	30-day mortality
Ko et al. (12)	Case-control study	8	Korea	51	2005–2018	52	35 (68.6%)	In-hospital mortality
Ko et al. (13)	Case-control study	7	Korea	48	2005–2011	53	33 (69%)	In-hospital mortality
Kofteridis et al. (14)	Cohort study	8	Greece	62	2004–2013	65	43 (69.35%)	In-hospital mortality
Korkmaz et al. (15)	Cohort study	7	Turkey	43	2009–2015	57	30 (69.8%)	30-day mortality
Lemiale et al. (16)	Case-control study	7	France	139	1988–2011	48	79 (56.8%)	ICU mortality
Li et al. (17)	Case-control study	8	Taiwan	20	2008–2011	50	9 (45%)	In-hospital mortality
Liu et al. (18)	Case-control study	8	China	57	2013–2018	48	29 (50.8%)	In-hospital mortality
Matsumura et al. (19)	Case-control study	7	Japan	82	2005–2010	64	51 (62%)	30-day mortality
Roblot et al. (20)	Case-control study	7	France	102	1995–1999	57	61 (59%)	30-day mortality
Tamai et al. (21)	Case-control study	8	Japan	29	2006–2012	59	14 (48%)	In-hospital mortality
Weng et al. (22)	Case-control study	8	China	82	2012–2015	53	34 (41.5%)	In-hospital mortality
Ye et al. (23)	Case-control study	8	China	47	2003–2012	49	21 (44.68%)	30-day mortality
Zahar et al. (24)	Case-control study	8	France	39	1989–1999	48	20 (52.2%)	30-day mortality

*ICU, intensive care unit; NA, not available; NOS, Newcastle-Ottawa Scale*.

### Statistical Analysis

RevMan software (version 5.3; Cochrane Collaboration, Oxford, UK) was used for the meta-analysis. For the categorical outcomes, the odds ratio (OR) was calculated as the effect size, and for the continuous outcomes, the mean difference (MD) was used as the effect size. Statistical heterogeneity in publications was determined using the *I*^2^ statistic. The fixed-effect model was chosen as the main analysis method since heterogeneity was confirmed as not statistically significant. At least five studies reported on the outcome of interest, all of which were two-sided, and the statistical significance was set at the 0.05 level.

## Results

### Literature Search

**Figure 1:**
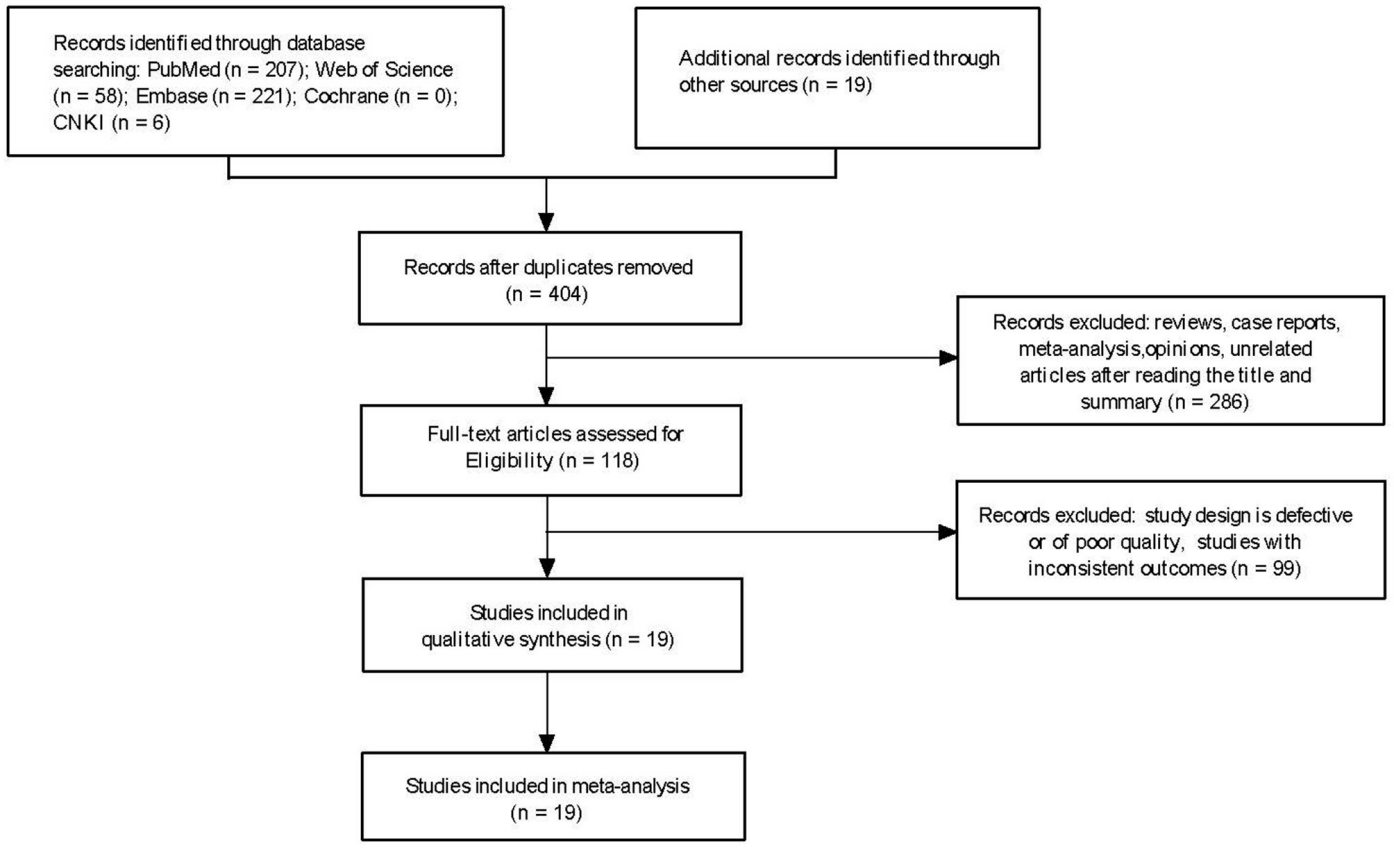
Flow diagram showing the study selection process.

A total of 404 papers were obtained for the initial screening. Finally, 19 articles, which met the inclusion criteria, were included (6–24). The 19 studies included 1,310 patients (485 and 825 in the non-survivor and survivor groups, respectively). The process of literature screening and selection are presented in Figure 1.

### Characteristics of the Articles Included and the Results of the Bias Risk Assessment

Table 1 details the basic features of the included articles and the results of the bias risk assessment according to the NOS. Of the 19 included studies, 17 used the case-control study design (6–13, 16–24) while two used a retrospective cohort design (14, 15). Four studies were performed in China (7, 18, 22, 23), four in Korea (10–13), four in Japan (6, 9, 19, 21), one in Taiwan (17), one in Turkey (15), one in Greece (14), one in Israel (8) and three in France (16, 20, 24). The sample sizes of these studies ranged from 20 to 173. More male patients were included (mean, 58%), and the mean age of the population ranged from 39 to 69 years.

Tables 2, 3 summarize the relevant information on the meta-analysis of modifiable and non-modifiable risk factors, respectively.

**Table 2:** Summary of meta-analysis results of non-modifiable factors for mortality in non-HIV-related *Pneumocystis* pneumonia.

**Factors**	**No. study**	**Sample (n)**	**Heterogeneity test**	**Effects model**	**OR/MD (95% CI)**	***P*-value**	
			***P*-value**	***I^**2**^* (%)**			
Age	14	990	0.24	20	Fixed effects model	6.61 (4.48–8.74)	<0.00001
Sex	13	948	0.21	23	Fixed effects model	1.02 (0.77–1.35)	0.9
Concomitant with other pulmonary diseases at diagnosis of PCP	6	477	0.52	0	Fixed effects model	3.42 (1.96–5.96)	<0.0001
Solid tumors	6	534	0.46	0	Fixed effects model	2.06 (1.32–3.21)	0.002

*CI, confidence interval; MD, mean difference; OR, odds ratio*.

**Table 3:** Summary of meta-analysis results of modifiable factors for mortality in non-HIV-related *Pneumocystis* pneumonia.

**Factors**	**No. study**	**Sample (*n*)**	**Heterogeneity test**	**Effects model**	**OR/MD (95% CI)**	***P*-value**	
			***P*-value**	**I^**2**^ (%)**			
Cytomegalovirus	5	287	0.21	32	Fixed effects model	3.59 (1.91–6.75)	<0.0001
Pneumothorax	6	340	0.45	0	Fixed effects model	2.55 (1.13–5.77)	0.02
PCP prophylaxis	7	640	0.67	0	Fixed effects model	1.43 (0.75–2.71)	0.28
Corticosteroid after admission	8	675	0.36	8	Fixed effects model	1.44 (0.94–2.22)	0.1
Invasive ventilation	12	849	0.01	54	Random effects model	29.24 (13.09–65.33)	<0.00001
Lactate dehydrogenase	9	538	0.002	67	Random effects model	91.12 (1.16–181.09)	0.05
Albumin	9	618	<0.00001	88	Random effects model	−0.18 (-0.46–0.10)	0.22
Lymphocyte count	10	711	0.03	51	Random effects model	−0.25 (-0.39−0.10)	0.0008
Time from onset of symptoms to treatment	6	499	0.007	69	Random effects model	1.9 (-1.18–4.99)	0.23

*CI, confidence interval; MD, mean difference; OR, odds ratio; PCP, Pneumocystis pneumonia*.

### Meta-Analysis Results

#### Non-modifiable Factors

**Figure 2:**
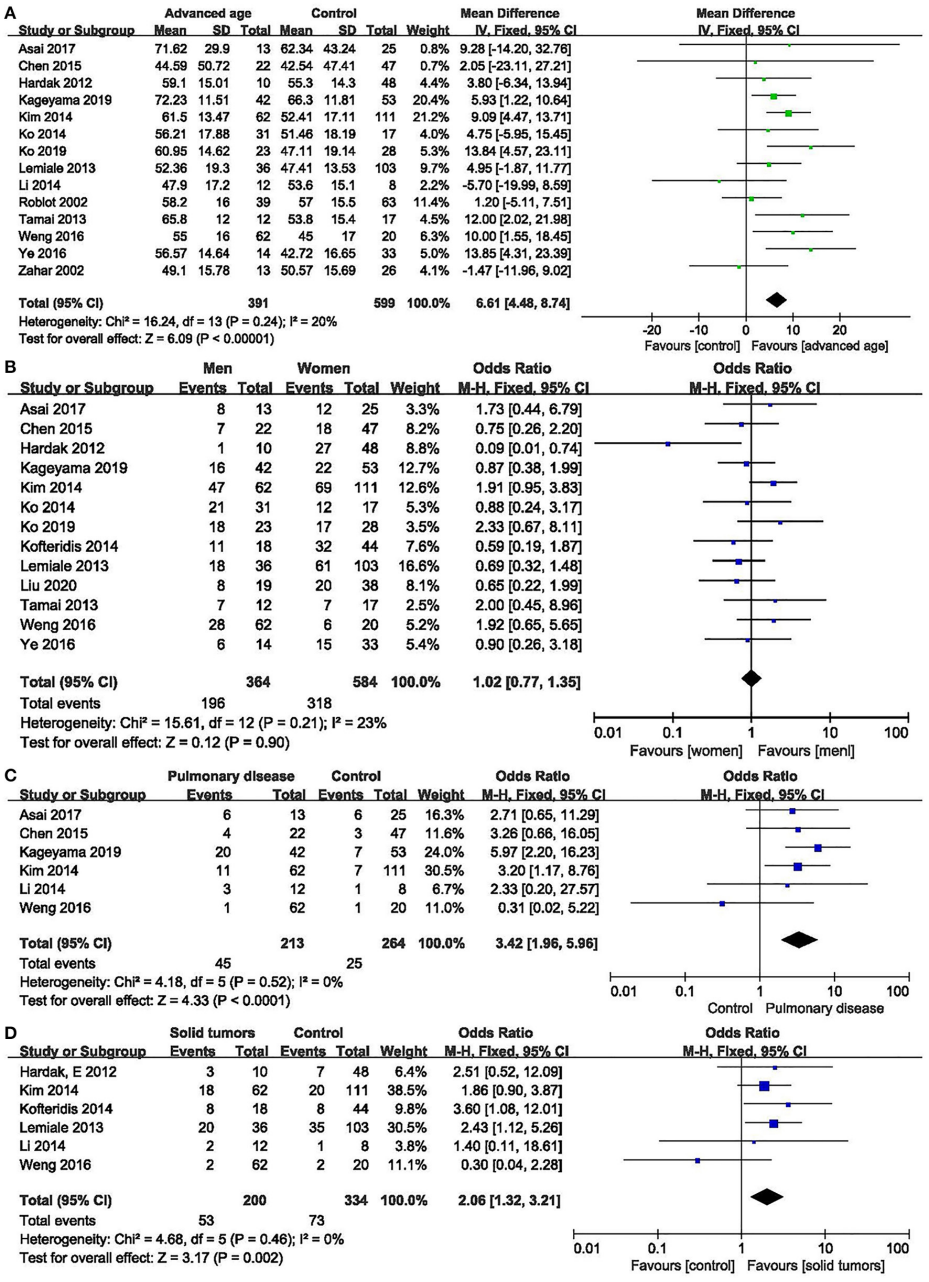
Non-modifiable factors. **(A)** Forest plots (fixed effects model) of meta-analysis on the association between age and the risk of mortality in non-HIV-related *Pneumocystis* pneumonia. **(B)** Forest plots (fixed effects model) of meta-analysis on the association between sex and the risk of mortality in non-HIV-related *Pneumocystis* pneumonia. **(C)** Forest plots (fixed effects model) of meta-analysis on the association between pulmonary disease and the risk of mortality in non-HIV-related *Pneumocystis* pneumonia. **(D)** Forest plots (fixed effects model) of meta-analysis on the association between solid tumors and the risk of mortality in non-HIV-related *Pneumocystis* pneumonia.

Age: fourteen studies (6–10, 12, 13, 16, 17, 20–24) were included, with 391 and 599 patients in the non-survivor and group, respectively. The results showed that age was a risk factor of mortality from PcP in non-HIV patients (mean difference = 6.61, 95% CI 4.48 to 8.74, *P* < 0.00001; Figure 2A). No significant heterogeneity was found in this analysis (*I*^2^ = 20%, *P* = 0.24).

Sex: thirteen studies (6–10, 12, 13, 15, 16, 18, 21–23) were included, with 364 and 584 patients in the non-survivor and survivor groups, respectively. The results showed that sex was not a risk factor of mortality from PcP in non-HIV patients (OR 1.02, 95% CI 0.77 to 1.35, *P* = 0.90; Figure 2B). No significant heterogeneity was found in this analysis (*I*^2^ = 23%, *P* = 0.21).

Concomitant with other pulmonary diseases at diagnosis of PcP: six studies (6, 7, 9, 10, 17, 22) were included, with 213 and 264 patients in the non-survivor and survivor groups, respectively. The Results showed that concomitant with other pulmonary diseases at diagnosis of PcP was a risk factor of mortality from PcP in non-HIV patients (OR 3.42, 95% CI, 1.96 to 5.96; *P* < 0.0001; Figure 2C). No significant heterogeneity was found in this analysis (*I*^2^ = 0%, *P* = 0.52).

Solid tumors:six studies (8, 10, 14, 16, 17, 22) were included, with 200 and 334 patients in the non-survivor and survivor groups, respectively. The results showed that solid tumors were a risk factor of mortality from PcP in non-HIV patients (OR 2.06, 95% CI, 1.32 to 3.21; *P* = 0.002; Figure 2D). No significant heterogeneity was found in this analysis (*I*^2^ = 0%, *P* = 0.46).

#### Modifiable Risk Factors

**Figure 3:**
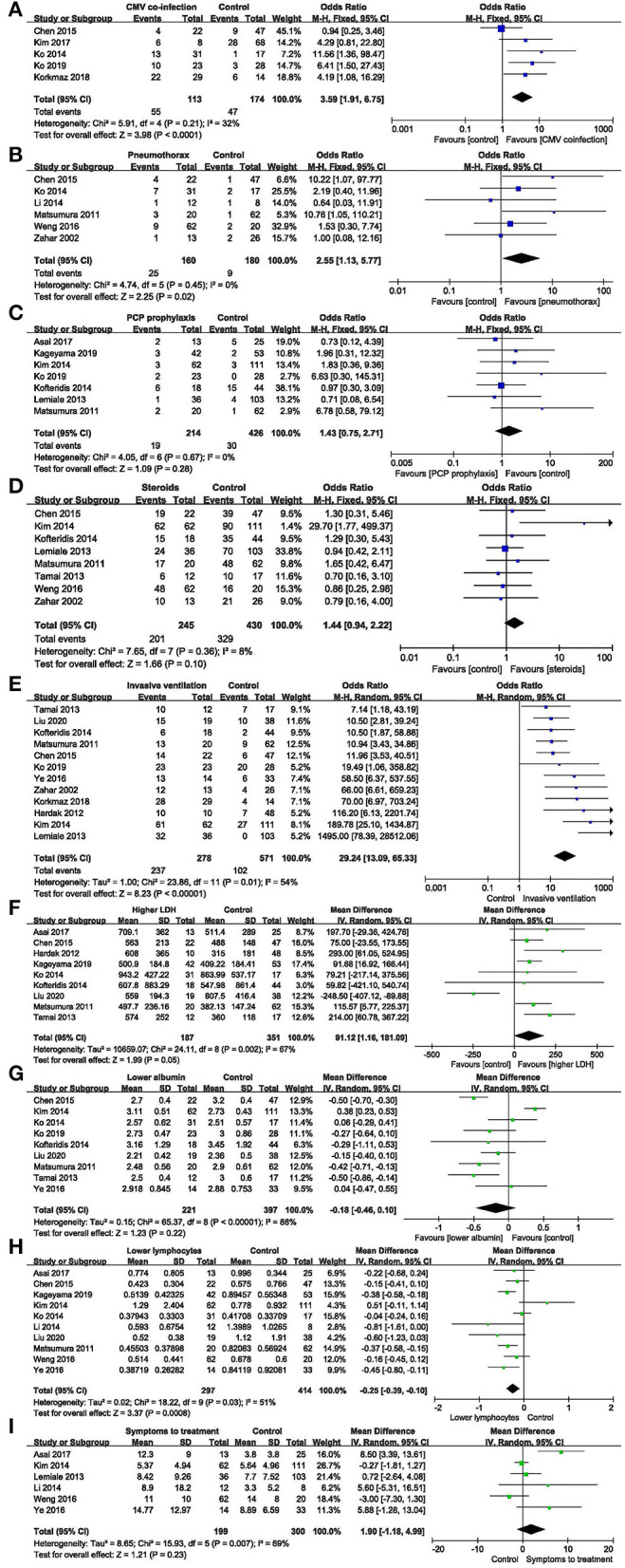
Modifiable factors. **(A)** Forest plots (fixed effects model) of meta-analysis on the association between cytomegalovirus co-infection and the risk of mortality in non-HIV-related *Pneumocystis* pneumonia. **(B)** Forest plots (fixed effects model) of meta-analysis on the association between pneumothorax and the risk of mortality in non-HIV-related *Pneumocystis* pneumonia. **(C)** Forest plots (fixed effects model) of meta-analysis on the association between prophylaxis and the risk of mortality in non-HIV-related *Pneumocystis* pneumonia. **(D)** Forest plots (fixed effects model) of meta-analysis on the association between the use of corticosteroids after admission and the risk of mortality in non-HIV-related *Pneumocystis* pneumonia. **(E)** Forest plots (random effects model) of meta-analysis on the association between invasive ventilation during hospitalization and the risk of mortality in non-HIV-related *Pneumocystis* pneumonia. **(F)** Forest plots (random effects model) of meta-analysis on the association between lactate dehydrogenase and the risk of mortality in non-HIV-related *Pneumocystis* pneumonia. **(G)** Forest plots (random effects model) of meta-analysis on the association between albumin and the risk of mortality in non-HIV-related *Pneumocystis* pneumonia. **(H)** Forest plots (random effects model) of meta-analysis on the association between lymphocytes and the risk of mortality in non-HIV-related *Pneumocystis* pneumonia. **(I)** Forest plots (random effects model) of meta-analysis on the association between time from onset of symptoms to treatment and the risk of mortality in non-HIV-related *Pneumocystis* pneumonia.

Cytomegalovirus (CMV) co-infection: five studies (7, 11–13, 15) were included, with 113 and 174 patients in the non-survivor and survivor groups, respectively. The results showed that lung disease was a risk factor of mortality from PcP in non-HIV patients (OR 3.59, 95% CI 1.91 to 6.75, *P* < 0.0001; Figure 3A). No significant heterogeneity was found in this analysis (*I*^2^ = 32%, *P* = 0.21).

Pneumothorax: six studies (7, 13, 17, 19, 22, 24) were included, with 160 and 180 patients in the non-survivor and survivor groups, respectively. The results showed that pneumothorax was a risk factor of mortality from PcP in non-HIV patients (OR 2.55, 95% CI 1.13 to 5.77, *P* = 0.02; Figure 3B). No significant heterogeneity was found in this analysis (*I*^2^ = 0%, *P* = 0.45).

PcP prophylaxis: seven studies (6, 9, 10, 12, 14, 16, 19) were included, with 214 and 426 patients in the non-survivor and survivor groups, respectively. The results showed that the association between PcP prophylaxis and mortality was insignificant (OR 1.43, 95% CI 0.75 to 2.71, *P* = 0.28; Figure 3C). No significant heterogeneity was found in this analysis (*I*^2^ = 0%, *P* = 0.67).

The use of corticosteroids after admission: eight studies (7, 10, 14, 16, 19, 21, 22, 24) were included, with 245 and 430 patients in the non-survivor and survivor groups, respectively. The results showed that the use of corticosteroids after admission was not a risk factor of mortality from PcP in non-HIV patients (OR 1.44, 95% CI, 0.94 to 2.22; *P* = 0.1; Figure 3D). No significant heterogeneity was found in this analysis (*I*^2^ = 8%, *P* = 0.36).

Invasive ventilation during hospitalization: twelve studies (7, 8, 10, 12, 14–16, 18, 19, 21, 23, 24) were included, with 278 and 571 patients in the non-survivor and survivor groups, respectively. After the heterogeneity test (*I*^2^ = 54%, *P* = 0.01), the random-effects model was used. The results showed that invasive ventilation during hospitalization was a risk factor of mortality from PcP in non-HIV patients (OR 29.24, 95% CI 13.09 to 65.33, *P* < 0.00001; Figure 3E).

Lactate dehydrogenase (LDH): nine studies (6–9, 13, 14, 18, 19, 21) were included, with 187 and 351 patients in the non-survivor and survivor groups, respectively. After the heterogeneity test (*I*^2^ = 67%, *P* = 0.002), the random effects model was used, and the results showed that LDH was a risk factor of mortality from PcP in non-HIV patients (mean difference = 91.12, 95% CI 1.16 to 181.09, *P* = 0.05; Figure 3F).

Albumin: nine studies (7, 10, 12–14, 18, 19, 21, 23) were included, with 221 and 397 patients in the non-survivor and survivor groups, respectively. After the heterogeneity test (*I*^2^ = 88%, *P* < 0.00001), the random effects model was used, and the results showed that albumin was not a risk factor of mortality from PcP in non-HIV patients (mean difference = −0.18, 95% CI −0.46 to 0.10, *P* = 0.22; Figure 3G).

Lymphocyte count: ten studies (6, 7, 9, 10, 13, 18, 22, 23) were included, with 297 and 414 patients in the non-survivor and survivor groups, respectively. After the heterogeneity test (*I*^2^ = 51%, *P* = 0.03), the random effects model was used, and the results showed that lymphocyte count was a risk factor of mortality from PcP in non-HIV patients (mean difference = −0.25, 95% CI −0.39 to −0.10, *P* = 0.0008; Figure 3H).

Time from onset of symptoms to treatment: six studies (6, 10, 16, 17, 22, 23) were included, with 199 and 300 patients in the non-survivor and survivor group, respectively. After the heterogeneity test (*I*^2^ = 69%, *P* = 0.007), the random-effects model was used, and the results showed that time from onset of symptoms to treatment was not a risk factor of mortality from PcP in non-HIV patients (mean difference = 1.90, 95% CI −1.18 to 4.99, *P* = 0.23; Figure 3I).

## Discussion

Both non-modifiable and modifiable risk factors can influence the mortality of PcP. Our meta-analysis systematically pooled 19 studies (including 1,310 non-HIV patients with PcP), which reported the prognostic factors in these patients. In the present study, age, concomitant with other pulmonary diseases at diagnosis of PcP, solid tumors, CMV co-infection, LDH, lymphocyte count, invasive ventilation during hospitalization, and pneumothorax were risk factors of mortality from non-HIV-related PcP. Some findings in our study were novel and deserve further discussion.

Advanced age is associated with a worse PcP prognosis in HIV-negative subjects (25–27). This study also found that age is a risk factor for mortality from PcP, which might be related to the progressive decline of somatic function with increasing age.

Our results revealed that in HIV-negative patients, PcP mortality was influenced by underlying pulmonary disease (refers to concomitant with other pulmonary diseases at diagnosis of PcP), and an increasing number of reports have described the pulmonary colonization of PcP in immunosuppressive patients with underlying pulmonary diseases (28, 29). A previous report indicated that up to 33.8% of patients with interstitial lung diseases are colonized with *pneumocystis* (30), which may contribute to the severity of the disease. Moreover, Enomoto et al. (31) conducted the first study focusing on HIV-negative PcP patients with underlying pulmonary diseases and found that all the five HIV-negative PcP patients with no underlying pulmonary disease survived, whereas nine of the 12 HIV-negative PcP patients with underlying pulmonary disease died. The current literature has not identified the mechanism by which pulmonary disease affects the prognosis of PcP. We assume that chronic pulmonary disease might lead to the destruction of the lung structure, making *pneumocystis* in the lungs more likely to progress, thereby increasing mortality.

The pooled results of this meta-analysis demonstrate that solid tumors are one of the comorbidities associated with an increased mortality rate in non-HIV PcP patients, which might be related to the immunosuppressive state, making it easier for pathogens to spread. New treatment of solid tumors, including more intensive chemotherapy, increases the survival rate of patients with malignancies. However, such advanced states increase the risk of treatment-related life-threatening complications, which leads to increased mortality (32, 33).

Our analysis of five articles found that CMV co-infection increased mortality in non-HIV PcP patients, although there was no significant difference in mortality among HIV patients with PcP based on their CMV infection status (34–37). In contrast to the above results, some previous researchers found that pulmonary CMV co-infection does not increase the risk of death (11, 38). Korkmaz et al. (15) found that the development of acute respiratory distress syndrome (ARDS) and the requirement for mechanical ventilation are more common in the CMV co-infection group, requiring a longer duration of stay in the intensive care unit (ICU), while CMV co-infection was not an independent risk factor in multivariate analysis for mortality. In kidney transplant recipients with PcP, mortality became doubled in the PcP combined with CMV group than that in the PcP only group, but the difference was not statistically significant (39). These differences in results might on account of the heterogeneity of HIV-negative patients in different studies, including different underlying diseases. Furthermore, the differences in the copy number of CMV between studies, reflecting whether CMV is active or pathogenic, might also relate to the heterogeneity of these results [higher CMV DNA load in the bronchoalveolar lavage fluid was positively associated with increased mortality (38)]. Therefore, further large-scale prospective studies are needed to determine whether CMV infection in non-HIV PcP patients affects the prognosis.

As expected, high serum LDH levels are a risk factor affecting the prognosis of PcP. LDH is considered as a predictive parameter of disease severity as well as a marker for assessing the condition of patients during treatment. It has also been found that higher LDH levels at PcP diagnosis is a risk factor for death (40). The serum LDH in PcP patients, which is released from the cells after the cytoplasmic membrane is damaged, might increase after the lung injury caused by the pathogen.

The combined analysis of 10 studies in the present study showed that a low lymphocyte count was associated with death. Most of the patients in our study were immunosuppressed, and lymphocyte count may be a helpful marker to determine the risk of PcP in immunosuppressed patients without HIV (40). Meanwhile, when infected with PcP in non-HIV patients, the lymphocyte count can also be used to predict mortality.

Based on our findings, invasive ventilation during hospitalization was associated with increased mortality. This finding is consistent with a previous study (41). Invasive ventilation often indicates a state of severe hypoxemia, and multiple organs are also likely to be in a state of failure or ARDS. Therefore, it is expected that the mortality rate will increase significantly, and this may be explained by ventilator-associated pneumonia. To correct hypoxia, the set ventilator parameters are generally high (26). This often leads to ventilator-associated lung injuries. Additional studies are required to identify approaches to improving the protective lung ventilation strategies for this population. Barotrauma, including pneumothorax, pneumomediastinum and pneumohypoderma, caused by invasive ventilation, can also explain the extremely poor prognosis (42). Pneumothorax is currently an unsolved problem in PcP. The development of pneumothorax is independently associated with increased mortality in PcP patients without HIV infection (43).

Hypoalbuminemia is reported as an independent risk factor of mortality in a study performed on HIV patients with PcP infection (44), and it has also been reported as an independent risk factor for non-HIV (45, 46). Albumin has antioxidant activity and can protect the cells from oxidative stress in sepsis. It is also an indicator of inflammation, suggesting the severity of the disease. Albumin levels decrease during acute and chronic inflammation since the cytokines inhibit its synthesis in the liver (47). However, our analysis showed no association between low serum albumin levels and mortality. That the results were statistically significant may be related to the relatively small sample size.

It is worrying that PcP prophylaxis was not generally administered in immunocompromised non-HIV patients. Our analysis showed that only 7.66% (49/640) of HIV-negative patients with PcP received appropriate prophylaxis before the onset of disease. No significant differences were observed in mortality between patients with or without prophylaxis in the current meta-analysis. This result might be affected by the degree of illness in different populations reported in different studies and the limited sample sizes, and therefore cannot be extrapolated. Prophylaxis is effective in susceptible individuals and is administered in most transplant centers. The American Society of Transplantation recommends general prophylaxis for 6–12 months after solid organ transplantation. If there is anti-rejection therapy, long-term neutropenia, or corticosteroid therapy with a dose of >20 mg prednisolone equivalent for more than 4 weeks, preventive measures should be extended. Lifelong prophylaxis is recommended for patients with chronic CMV infection, a history of PcP infection, or after the lung or small-bowel transplantation (48). The Kidney Disease Improving Global Outcomes (KDIGO) guidelines recommend prophylaxis with trimethoprim/sulfamethoxazole daily for 3–6 months, and for 6 weeks after anti-rejection therapy in kidney transplant patients (49). In summary, PcP prevention is necessary.

The use of adjunctive steroids during hospitalization may not improve PcP outcomes in non-HIV patients. For HIV-negative patients with PcP, there is no strong evidence that these patients may benefit from adjunctive steroids (50). Two previous studies (51, 52) analyzed the effects of steroids on non-HIV patients with severe PcP (PaO2 < 70 mmHg) and reported that adjunctive steroids failed to reduce hospital mortality. Another study found that high-dose steroid treatment (≥1 mg/kg/day prednisone equivalent) was an independent predictor of ICU mortality (51). However, some factors may have an impact on the role of adjunctive steroids in mortality, such as the dose of adjunctive steroids, severity of the disease, whether adjunctive steroids are added for the treatment of PcP, and whether the patient has used adjunctive steroids to treat the primary disease before the PcP. These factors were mixed in the included studies, and this question needs to be answered by a well-designed large randomized clinical trial. Therefore, the decision to add steroids as an adjunctive treatment should be individualized.

Early diagnosis and empirical treatment initiation could improve mortality in non-HIV-PcP patients (16, 53, 54). Treatment delay has previously been considered as a factor associated with poor prognosis in patients with PcP (55). However, this meta-analysis did not find time from symptom onset to treatment was an independent poor prognostic factor for non-HIV PcP. There are many confounding factors, including the large top tertiary hospitals we had included. Many of the patients admitted to these tertiary hospitals were referral patients who were financially capable or in critical condition, probably resulting in the mixed conclusions. We believe that more patients with the same basic characteristics are required to further explore this finding.

## Limitation

There are some limitations to the present meta-analysis. Firstly, our meta-analysis is based on observational data, and therefore, the results are not as robust as those of randomized controlled trials. Secondly, the characteristics of the included patients in different studies differed, including the methods of diagnosis of PcP, sample selection, and follow-up, which might contribute to the statistical and clinical heterogeneity. Moreover, the limited sample size may also influence the application of the results. Thirdly, only English language studies were included, and this review did not include unpublished researches or those published in the “gray literature.” Thus, differences about region, race and other influencing factors may not been fully reflected.

## Conclusion

The mortality rate of non-HIV-infected patients with PcP was still high. Although high-level evidence is lacking, this study demonstrates that age, concomitant with other pulmonary diseases at diagnosis of PcP, solid tumors, CMV co-infection, LDH, lymphocyte count, invasive ventilation during hospitalization, and pneumothorax are risk factors of mortality from non-HIV-related *Pneumocystis* pneumonia. However, data supporting several other indices are insufficient. Hence, further large-scale studies are required to verify these findings. Improved knowledge of prognostic factors is crucial to guide early treatment.

## Data Availability Statement

The original contributions presented in the study are included in the article/supplementary materials. Further inquiries can be directed to the corresponding author.

## Author Contributions

QZ was involved in the administrative support, conception and design of the manuscript, data interpretation, reviewed, and revised the manuscript. YW was involved in the conception and design of the manuscript, collection and assembly of the data, data analysis, and drafted the initial manuscript. XZ and MS was involved in the collection and assembly of data and drafted the initial manuscript. XH was involved in the conception and design of the manuscript, reviewed, and revised the manuscript. TS was involved in the conception, design of the manuscript, and data interpretation. GF was involved in the critically reviewed the manuscript for important intellectual content. All authors contributed to the article and approved the submitted version.

## Conflict of Interest

The authors declare that the research was conducted in the absence of any commercial or financial relationships that could be construed as a potential conflict of interest.
